# Vitamin D Deficiency is not Associated with Higher Levels of SYNTAX Score

**DOI:** 10.21470/1678-9741-2018-0178

**Published:** 2019

**Authors:** Levent Cerit, Zeynep Cerit

**Affiliations:** 1 Department of Cardiology, Near East University, Faculty of Medicine, Nicosia, Cyprus.; 2 Department of Pediatric Cardiology, Near East University, Nicosia, Cyprus.

**Keywords:** Vitamin D, Risk Assessment, Hypertension, Diabetes Mellitus, Hyperlipidemia

## Abstract

**Objective:**

To evaluate the association between serum vitamin D (vitD) level and SYNTAX (synergy between percutaneous coronary intervention with taxus and cardiac surgery) score (SS).

**Methods:**

The medical records of consecutive patients, who underwent coronary artery bypass graft surgery, were retrospectively reviewed. The study group consisted of 158 patients. Biochemical, clinical, and echocardiographic parameters and SS were evaluated in all patients. The patients were divided into 2 groups according to SS (≥23= high, <23= low).

**Results:**

The high SYNTAX score (HSS) group was older and had higher body mass index, C-reactive protein (CRP), low-density lipoprotein, and fasting plasma glucose level than the low SYNTAX score (LSS) group. The HSS group had lower high-density lipoprotein and vitD level than the LSS group. The HSS group had a higher prevalence of diabetes mellitus (DM), hypertension (HT), hyperlipidemia (HL), and current smoking patients than the LSS group. On univariate analysis, age, HT, DM, HL, smoking, CRP, and serum vitD level were associated with HSS. On multivariate analysis, HT, DM, and HL were independent predictors of HSS (odds ratio [OR]: 2.137, 95% confidence interval [CI]: 1.468-2.935, *P*<0.001; OR: 3.559, 95% CI: 2.763-5.927, *P*<0.001; OR: 2.631, 95% CI: 1.529-3.438, *P*<0.001; respectively).

**Conclusion:**

In our study, we have found out that HT, DM, and HL were independent predictors of HSS. Serum vitD level was not found to be an independent predictor of HSS.

**Table:** 

Abbreviations, acronyms & symbols				
ACE-I	= Angiotensin-converting enzyme inhibitor		HSS	= High SYNTAX score
ARB	= Angiotensin-receptor blocker		HT	= Hypertension
CABG	= Coronary artery bypass graft		LSS	= Low SYNTAX score
CAD	= Coronary artery disease		OR	= Odds ratio
CI	= Confidence interval		ROC	= Receiver operating characteristic
CRP	= C-reactive protein		SD	= Standard deviation
DM	= Diabetes mellitus		SS	= SYNTAX score
FEV1	= Forced expiratory volume in 1 second		SYNTAX	= Synergy between percutaneous coronary intervention with taxus and cardiac surgery
FVC	= Forced vital capacity		vitD	= Vitamin D
HL	= Hyperlipidemia			

## INTRODUCTION

Vitamin D (vitD) has well-established roles in calcium and bone health. VitD receptors are found in the brain, cardiomyocytes, vascular smooth muscle cells, endothelial cells, pancreatic beta-cells, skeletal muscle, and macrophages. VitD deficiency is independently associated with cardiovascular morbidity and mortality in the general population^[1]^. Growing evidence demonstrated that vitD deficiency is closely associated with hypertension (HT), obesity, diabetes mellitus (DM), metabolic syndrome, and inflammation^[2-6]^. VitD deficiency plays a key role in the pathogenesis of plaque formation via endothelial dysfunction, activation of the renin-angiotensin system, and inflammation^[7-9]^.

SYNTAX (synergy between percutaneous coronary intervention with taxus and cardiac surgery) score (SS) is an angiographic grading tool to evaluate the complexity and extensity of coronary artery disease (CAD). It is widely used for determining the optimal revascularization strategy. It is also a powerful stratification mechanism allowing uniform, standardised assessment of CAD extensity and severity^[10-13]^. Previous studies demonstrated that vitD deficiency is an independent predictor of high SYNTAX score (HSS) in patients undergoing coronary angiography. In a study conducted by Chen et al.^[14]^, 348 consecutive patients who underwent coronary angiography for evaluation of CAD were included. Lower serum vitD level was found to be an independent predictor of HSS. In the same manner, Şeker et al.^[15]^ reported that serum vitD level independently associated with SS.

There is scarce data about the association between vitD and SS in patients who underwent coronary artery bypass graft (CABG) surgery. In the light of this knowledge, we assessed the relationship between serum vitD level and SS in patients who underwent CABG surgery.

## METHODS

This is a cross-sectional study. Our study was registered in the Research Registry website (research registry 4304). All procedures were performed in accordance with the Declaration of Helsinki. The STROBE guidelines were used to ensure the reporting of this observational study. The study was approved by the local ethics committee.

The study group consisted of 158 consecutive patients who underwent on-pump CABG surgery. A retrospective evaluation of consecutive CABG patients was performed. Patients with chronic liver disease, heart failure, chronic renal failure, obstructive sleep apnoea, chronic obstructive pulmonary disease, inflammatory diseases, bone disorders, systemic infection, acute coronary syndromes, and thyroid disorders were excluded. If the patients were taking cholecalciferol or vitamin D3 tablets they were excluded. Thirteen patients were excluded according to the exclusion criteria. A total of 145 patients were included in our study.

The patients' data were retrospectively analyzed for demographic features, echocardiographic parameters, biochemical parameters, and SS.

### Echocardiographic Examination

All patients underwent transthoracic echocardiography using Vivid S5 (GE Healthcare) echocardiography device and Mass S5 probe (2-4 MHz). Standard two-dimensional and colour flow Doppler views were acquired according to the guidelines of the American Society of Echocardiography and the European Society of Echocardiography^[16]^. The ejection fraction was measured according to the Simpson's method.

### Coronary Angiography

All patients underwent elective coronary angiography according to the Judkins technique. Angiograms were reviewed by at least 2 non-blinded reviewing cardiologists.

SYNTAX Score

All lesions causing ≥50% of stenosis in a coronary artery with a diameter ≥1.5 mm were included in the SS calculation. For calculation, the website software (http://www.SYNTAXscore.com) was used. Scoring was performed for each patient in keeping with the following parameters: coronary dominance, number of lesions, segments included per lesion, the presence of total occlusion, bifurcation, trifurcation, aorto-osteal lesion, severe tortuosity, calcification, thrombus, diffuse/small vessel disease, and lesion length >20 mm. SS was evaluated separately by 2 interventional cardiologists blinded to the study protocol and patients' characteristics. In the presence of a contradiction between the 2 results, the opinion of a senior interventional cardiologist was applied, and a common consensus was obtained. SS was divided into 2 groups: ≥23= high, <23= low.

### Blood Samples

Fasting venous blood samples were obtained from all patients following a fasting period of 8 hours to determine laboratory parameters. Serum 25-(OH) vitD levels were measured by chemiluminescent immunoassay using a LIAISON analyser (DiaSorin Inc). VitD deficiency was defined as serum level of 25-(OH) vitD <20 ng/ml.

Patients with DM were identified on admission as those with documented DM using either oral hypoglycemic agents or insulin treatment. HT was defined as blood pressure >140/90 mmHg or use of antihypertensive therapy on admission. Hyperlipidemia (HL) was defined as total cholesterol <200 mg/dl or use of antihyperlipidemic therapy on admission. Chronic obstructive pulmonary disease was defined as forced expiratory volume in 1 second/forced vital capacity (FEV1/FVC) <70% and FEV1 >80% of predicted value. Heart failure diagnosis was based on clinical features and echocardiographic results. Patients with clinical features of heart failure or whose left ventricular ejection fraction were <50% were excluded from the study. Renal failure was defined as a serum creatinine level >1.5 mg/dl.

### Statistical Analysis

Statistical analysis was performed using the SPSS (version 20.0, SPSS Inc., Chicago, Il, USA) software package. Continuous variables were expressed as the mean ± standard deviation (mean±SD) and categorical variables were expressed as percentage (%). The Kolmogorov-Smirnov test was used to evaluate the variables distribution. Student's t-test was used to evaluate continuous variables showing normal distribution and Mann-Whitney U-test was used to evaluate variables that did not show normal distribution. A *P*-value <0.05 was considered statistically significant. To identify predictors of HSS, the following variables were initially assessed in a univariate model: age, HT, DM, HL, smoking, C-reactive protein (CRP), and vitD. Significant variables in univariate analysis were then entered into a multivariate logistic regression analysis using backwards stepwise selection.

## RESULTS

A comparison between the subjects' baseline characteristics is shown in Table 1. The mean age of the study group was 67.8 years, and 55.8% of the patients were female. The prevalence of vitD deficiency was found in 78.5% of the HSS group and 34% of the low SYNTAX score (LSS) group (Table 1).

**Table 1:** Patients' characteristics.

Patients' characteristics	SYNTAX score ≥23 (n=98)	SYNTAX score <23 (n=47)	*P*
Age (years)	74.9±10.7	61.3±8.4	<0.001
Female gender (%)	56.1	55.3	0.762
Body mass index (kg/m^2^)	27.8	24.1	0.04
Hypertension (%)	82.9	52.7	<0.001
Diabetes mellitus (%)	71.3	33.9	<0.001
Hyperlipidemia (%)	69.7	39.3	<0.001
Current smoking (%)	61.3	24.5	<0.001
Beta-blocker therapy (%)	88	90	0.624
Statin therapy (%)	78	81	0.839
ACE-I/ARB therapy (%)	74	71	0.719
Aspirin therapy (%)	97	94	0.627

ACE-I=angiotensin-converting enzyme inhibitor; ARB=angiotensin-receptor blocker; SYNTAX=synergy between percutaneous coronary intervention with taxus and cardiac surgery

The HSS group was older and had higher body mass index, CRP, low-density lipoprotein, and fasting plasma glucose level than the LSS group. The HSS group had lower high-density lipoprotein and vitD level than the LSS group. The HSS group had a higher prevalence of DM, HT, HL, and current smoking patients than the LSS group. There was no significant difference between the 2 groups regarding female gender, beta-blocker, statin, aspirin, and angiotensin-converting enzyme inhibitor/angiotensin-receptor blocker (ACE-I/ARB) therapy (Tables 1 and 2).

**Table 2:** Laboratory and echocardiographic parameters of the study population.

Laboratory parameters	SYNTAX score ≥23 (n=98)	SYNTAX score <23 (n=98)	*P*
Hemoglobin (g/dl)	13.7±3.4 (14.9)	14.1±2.7 (14.7)	0.729
Platelet (10^3^/µl)	299.3±31.6 (312.9)	293.7±28.6 (309.7)	0.638
White blood cells (10^3^/µl)	6.3±3.1 (7.9)	5.8±2.9 (8.1)	0.593
Creatinine (mg/dl)	0.97±0.23 (1.03)	0.89±0.18 (0.99)	0.624
Fasting plasma glucose (mg/dl)	110.7±24.3 (107.9)	96.3±32.7 (96.3)	0.02
C-reactive protein (mg/dl)	1.67±0.89 (1.83)	1.37±0.72 (1.47)	0.03
Total cholesterol (mg/dl)	193.5±37.1 (184.6)	186.9±34.8 (182.7)	0.761
High-density lipoprotein (mg/dl)	33.3±8.7 (34.6)	40.7±9.5 (42.3)	0.03
Low-density lipoprotein (mg/dl)	193.5±51.3 (201.4)	171.6±38.5 (176.1)	0.02
Triglyceride (mg/dl)	176.8.1±51.7 (186.9)	179.3±47.8 (189.3)	0.537
Vitamin D level (ng/ml)	13.9 ± 6.7	23.9 ± 4.7	<0.001
Vitamin D deficiency (<20 ng/ml) (n,%)	77 (78.5)	16 (34.0)	<0.001
Left ventricular ejection fraction (%)	60.9±4.1	62.5±3.7	0.731

SYNTAX=synergy between percutaneous coronary intervention with taxus and cardiac surgery

**Fig. 1:**
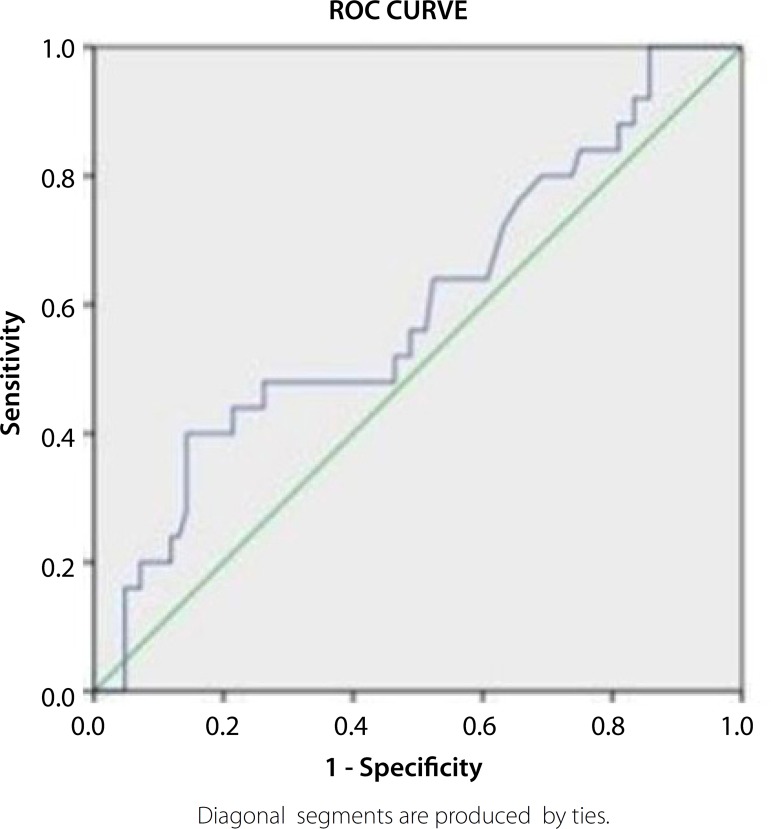
Receiver operating characteristic (ROC) curve for vitamin D for predicting high SYNTAX (synergy between percutaneous coronary intervention with taxus and cardiac surgery) score

On univariate analysis, age, HT, DM, HL, smoking, CRP, and vitD level were associated with HSS (Table 3). On multivariate analysis HT, DM, and HL were independent predictors of HSS (odds ratio [OR]: 2.137, 95% confidence interval [CI]: 1.468-2.935, *P*<0.001; OR: 3.559, 95% CI: 2.763-5.927, *P*<0.001; OR: 2.631, 95% CI: 1.529-3.438, *P*<0.001; respectively) (Table 4). Additionally, it was determined a cut-off level of 13.950 ng/ml for vitD for predicting HSS with a sensitivity of 80% and a specificity of 69% in receiver operating characteristic (ROC) curve analysis (area under the curve: 0.597, 95% CI: 0.468-0.727; Figure 1).

**Table 3:** Univariate analysis of predictors of high SYNTAX score.

Predictor variables	OR (95% CI)	*P*
Age (years)	1.679 (1.293-2.137)	<0.001
Hypertension, n (%)	2.761 (1.934-4.935)	<0.001
Diabetes mellitus, n (%)	3.437 (2.345-5.361)	<0.001
Hyperlipidemia, n (%)	2.359 (1.963-3.937)	<0.001
Current smoking, n (%)	1.738 (1.341-2.837)	<0.001
C-reactive protein, n (%)	1.914 (1.236-2.831	<0.001
Vitamin D (ng/ml)	2.318 (1.457-3.182)	<0.001

CI=confidence interval; OR=odds ratio; SYNTAX=synergy between percutaneous coronary intervention with taxus and cardiac surgery

**Table 4:** Multivariate analysis of predictors of high SYNTAX score.

Predictor variables	OR (95% CI)	*P*
Hypertension, n (%)	2.137 (1.468-2.935)	<0.001
Diabetes mellitus, n (%)	3.559 (2.763-5.927)	<0.001
Hyperlipidemia, n (%)	2.631 (1.529-3.438)	<0.001

CI=confidence interval; OR=odds ratio; SYNTAX=synergy between percutaneous coronary intervention with taxus and cardiac surgery

## DISCUSSION

In our study, we have found out that HT, DM, and HL were independent predictors of HSS. Serum vitD level was not found to be an independent predictor of HSS.

VitD deficiency is independently associated with cardiovascular morbidity and mortality in the general population^[1]^. Growing evidence demonstrates that vitD deficiency is strongly associated with various cardiovascular diseases, including postoperative atrial fibrillation and increased frequencies of HT, DM, obesity, HL, metabolic syndrome, and inflammation^[2-6,17,18]^. Several potential mechanisms established associations between vitD and atherosclerotic process. VitD may modulate endothelial cell function by decreasing the expression of adhesion molecules^[19]^. VitD deficiency might activate the renin-angiotensin system and promote oxidative stress^[8,20]^. Also, vitD deficiency may trigger an inflammatory process; it's well-known that inflammation is involved in the pathogenesis of all atherosclerotic stages^[21]^. Several studies found out that lower serum vitD level was an independent predictor of HSS^[14,15]^.

On the other hand, Pilz et al.^[22]^ conducted a larger cross-sectional study. They have evaluated 3299 Caucasian patients who were routinely referred to coronary angiography. They have reported that a low vitD status was not closely associated with prevalent CAD and that 25(OH)D and 1,25(OH)2D were not significantly associated with fatal myocardial infarction after multivariable adjustments. In the same manner, vitD was not found to be an independent predictor of HSS in our study.

HT, DM, and HL are major risk factors for the development of CAD and are associated with poor clinical outcomes. Changes in carbohydrate and lipid metabolism accompanying insulin resistance lead to the appearance of atherogenic lipoproteins, hyperglycemia, and increased the concentration of free fatty acids. Several studies found out that HT, DM, and HL are strongly associated with the extensity and complexity of CAD^[22,23]^. Our study showed that HT, DM, and HL are independent predictors of HSS, which is consistent with previous studies.

There are inconsistent data about the association between serum vitD level and CAD in literature. And there are scarce data about the association between vitD deficiency and SS in patients who underwent CABG surgery. In our study, we found out that HT, DM, and HL were independent predictors of HSS. Serum vitD level was not found to be an independent predictor of HSS.

Considering that both vitD deficiency and CAD have the same predisposing factors, including obesity, smoking, and sedentary lifestyle, our results suggest that the close association between vitD and CAD might be related with coexistence, rather than causality. In the light of this knowledge, we would like to emphasise that there must be strong evidence before hold the vitD deficiency accountable for the CAD severity. The questions to be asked are: Is there causality? Or just coexistence? Further studies with a larger number of patients are needed on this topic.

### Limitations of the Study

Our study has some limitations: first, the retrospective study design; second, a small sample size; third, we had no data about the patients' sun exposure time; and fourth, we had no data about patients' serum parathormone and calcium levels.

## CONCLUSION

In our study, we have found out that HT, DM, and HL were independent predictors of HSS. Serum vitD level was not found to be an independent predictor of HSS. Further studies with a larger number of patients are required for the evaluation of the association between serum vitD level and HSS.

**Table:** 

Authors' roles & responsibilities	
LC	Analysis and interpretation of data; drafting the paper; revising the work; approval of the final version
ZC	Analysis and interpretation of data; drafting the paper; revising the work; approval of the final version
